# A commentary of factors related to player availability and its influence on performance in elite team sports

**DOI:** 10.3389/fspor.2022.1077934

**Published:** 2023-01-16

**Authors:** Julio Calleja-González, Javier Mallo, Francesc Cos, Jaime Sampaio, Margaret T. Jones, Diego Marqués-Jiménez, Juan Mielgo-Ayuso, Tomás T. Freitas, Pedro E. Alcaraz, Javier Vilamitjana, Sergio J. Ibañez, Francesco Cuzzolin, Nicolás Terrados, Stephen P. Bird, Asier Zubillaga, Thomas Huyghe, Igor Jukic, Alberto Lorenzo, Irineu Loturco, Anne Delextrat, Xavi Schelling, Miguel Gómez-Ruano, Isaac López-laval, Jairo Vazquez, Daniele Conte, Álvaro Velarde-Sotres, Antonio Bores, Davide Ferioli, Franc García, Xavier Peirau, Rafael Martin-Acero, Carlos Lago-Peñas

**Affiliations:** ^1^ Department of Physical Education and Sports, Faculty of Education and Sport, University of the Basque Country, (UPV/EHU), Vitoria-Gasteiz, Spain; ^2^ Faculty of Kinesiology, University of Zagreb, Zagreb, Croatia; ^3^ Strength and Conditioning Society, Rome, Italy; ^4^ Facultad de Ciencias de la Actividad Física y del Deporte (INEF), Universidad Politécnica de Madrid, Madrid, Spain; ^5^ Manchester City Football Club 1^st^ Team, Manchester City, United Kingdom; ^6^ National Institute of Physical Education of Catalonia (INEFC), University of Barcelona, Barcelona, Spain; ^7^ Research Center in Sports Sciences, Health Sciences and Human Development, CIDESD, University of Trás-os-Montes e Alto Douro, UTAD, Vila Real, Portugal; ^8^ School of Kinesiology, George Mason University, Manassas, Virginia, VA, United States; ^9^ Valoración del rendimiento deportivo, actividad física y salud y lesiones deportivas (REDAFLED), Department of Didactics of Musical, Plastic and Corporal Expression, Faculty of Education, University of Valladolid, Soria, Spain; ^10^ Department of Health Sciences, Faculty of Health Sciences, University of Burgos, Burgos, Spain; ^11^ UCAM Research Center for High Performance Sport, Catholic University San Antonio, Murcia, Spain; ^12^ NAR - Nucleus of High Performance in Sport, São Paulo, Brazil; ^13^ Faculty of Sport Sciences, Catholic University of Murcia, Murcia, Spain; ^14^ Soccer Research Group, Friends Club - CDA, Buenos Aires, Argentina; ^15^ Group for Optimization of Training and Sport Performance (GOERD), Faculty of Sport Science, University of Extremadura, Cáceres, Spain; ^16^ University of Udine, Udine, Italy; ^17^ Regional Unit of Sports Medicine and Health Research Institute of the Principality of Asturias (ISPA), Oviedo, Spain; ^18^ School of Health and Medical Sciences Ipswich, Queensland, QLD, Australia; ^19^ Health Sciences and Social Work, Oxford Brookes University, Oxford, United Kingdom; ^20^ Institute of Sport, Exercise and Active Living, College of Sport and Exercise Science, Victoria University, Melbourne, VIC, Australia; ^21^ Faculty of Health and Sport Sciences, University of Zaragoza, Huesca, Spain; ^22^ Sport Performance Area, Fútbol Club Barcelona, Barcelona, Spain; ^23^ Institute of Sport Science and Innovations, Lithuanian Sports University, Kaunas, Lithuania; ^24^ Department of Movement, Human and Health Sciences, University of Rome “Foro Italico”, Rome, Italy; ^25^ Facultad de Ciencias de la Salud, Universidad Europea del Atlántico, Santander, Spain; ^26^ Departamento de Salud, Universidad Internacional Iberoamericana, Campeche, México; ^27^ Research Group Into Human Movement, Institut Nacional d'Educació Física de Catalunya (INEFC), Lleida, Spain; ^28^ Grupo de Aprendizaje y Control del Movimiento Humano. Facultade de Ciencias do Deporte e a Educación Física. Universidade da Coruña. Oleiros, A Coruña, Spain; ^29^ Faculty of Education and Sport Sciences, Governance and Economics Research Network, University of Vigo, Pontevedra, Spain

**Keywords:** team sport, performance, competition, recovery, training load

## Introduction

Elite performance and sporting success are often the result of optimal integration and synergy of all components of sports preparedness (i.e., health, technical and tactical skills, bioenergetic and neuromuscular abilities and capacities, anthropometric characteristics, cognition, emotions, creativity, or personality), which evolve because of systematic long-term sports preparation. However, the relative importance of these characteristics varies between individual and team sports. While some individual sports require a high standard of bioenergetic and neuromuscular abilities and capacities, team sports performance is closely related to technical and tactical skills, which may compensate for weakness within the fitness level (1). Nonetheless, successful team sport performances seem to be much more dependent on the interaction among a wide range of factors than on the maximum development of one or two factors in isolation. In team sports, elite performance emerges from the interaction among the individual parts (2) to overcome the opponent during competition.

Sports may be categorized according to the degree of predictability of the environment that they are played in (3). Team sports occur in highly unpredictable environments due to the interactions with both teammates and opponents, with performance dealing with this unpredictability. Thus, it is important to have a clear understanding of the integrative systems and the principles that rule their interactions with the environment, keeping in mind the main aim of the process: developing the diversity/unpredictability potential of athletes/teams (4) to afford the emergence of rich patterns of behavior from players to adapt quickly and effectively in dynamically changing and unpredictable environments (5).

**Figure 1:**
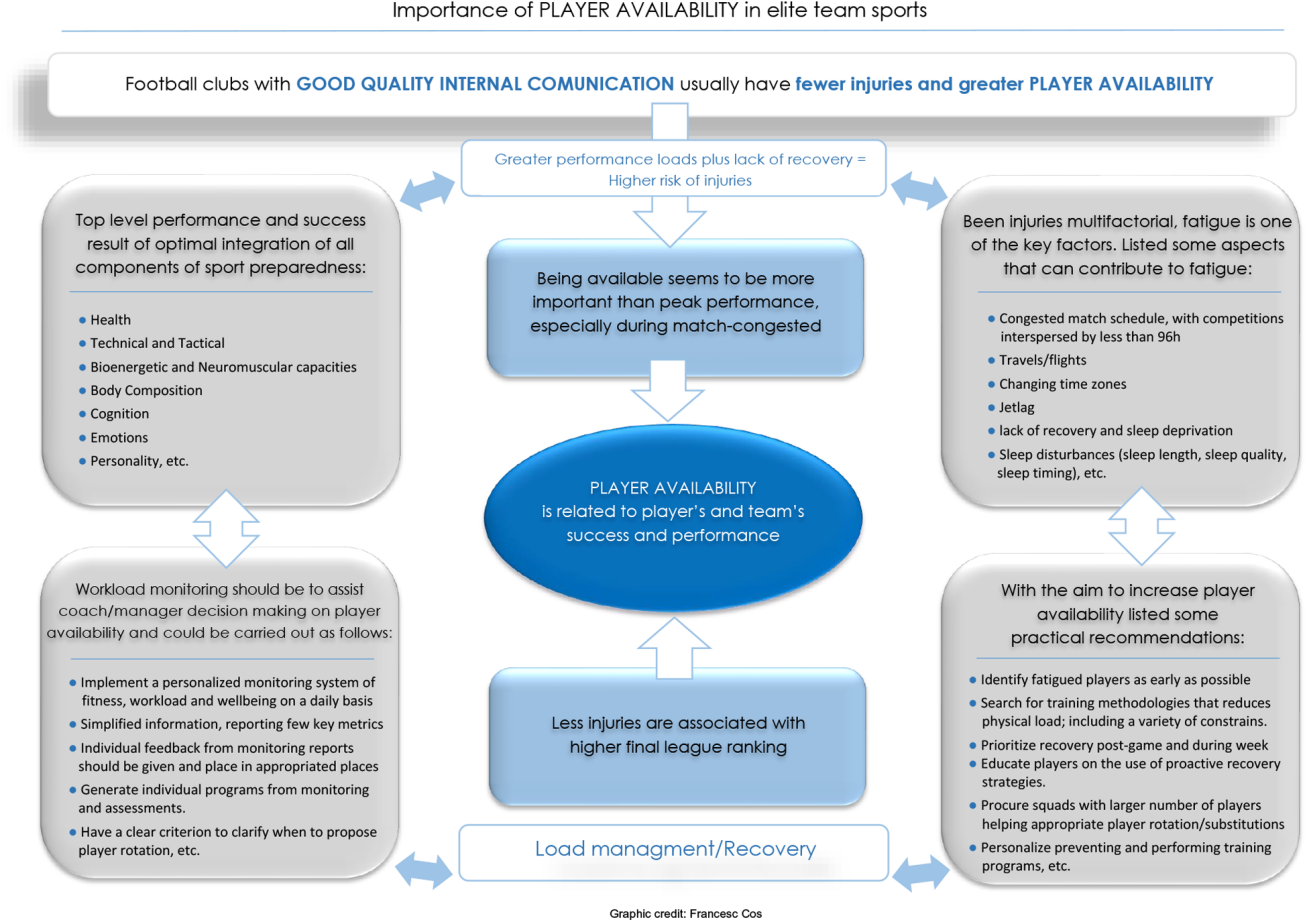
Importance of PLAYER AVAILABILITY in elite team sports.

Performance in team sports is affected by several factors that affect the organization of training and competitions. These include, for example, COVID-19 cases (6), PCR tests (7), air flights and their effects prior to competition (8), injuries (9), or match-congested schedules (10). The interaction among these factors may also influence player availability. The concept of player availability is a common one in elite team sports. Available players can be considered the ones who are injury-free and ready to compete whether the head coach chooses to put them on the lineup. Thus, an available state would be when a player is fit and recovered enough to compete. On the other hand, player unavailability would be considered a state which includes injury, sanction or suspension, or other reasons that would keep a player out of match. However, this topic needs to be explored more in elite team sport environments. Considering previous enriching work, it remains important to further progress and provide academic knowledge in order to support coaches/managers, strength and conditioning coaches, sport scientists, and medical team members (e.g., doctors, physicians, and physiotherapists) in their working environments. While widely-advocated scientific groundwork is considered throughout this manuscript, the main aim of this opinion article is to provide a review of factors related to player availability and its influence on performance in elite team sports (Figure 1). Finally, some practical suggestions and recommendations are provided to deal with constant alterations in player's availability and performance fluctuations.

## Match-congested schedules, performance and player availability

Elite team sports usually face match-congested schedules (i.e., many matches over short periods of time) during the preseason and the competitive season where the teams can be involved in two or more competitions simultaneously (e.g., national competition, continental competition) (10, 11). For instance, in South America, some Argentinian and Brazilian soccer teams must face a high frequency of competition (e.g., domestic tournament and Libertadores Cup) (12). These congested fixtures have been suggested to affect acute performance and well-being in different team sports, such as basketball (13, 14), soccer (15), futsal (16) or volleyball (17).

Team performance may be considerably affected by player availability (18). Thus, during the match-congested period the priority is to have the players available to compete (particularly remaining injury free), even if players are not at their individual peak of performance. Notwithstanding, the bioenergetic and neuromuscular abilities and capacities of the players should enable them to meet the match demands of competitive situations (e.g., most demanding scenario sequences) without endangering their health. Interestingly, in a recent systematic review and meta-analysis investigating the effects of match-congested schedules on performance in soccer, it was suggested that players may employ pacing strategies to maintain their high-intensity actions (19).

From the teamś perspective, managers could create squads with larger number of players to deal with (and complete) these ﬁxtures distribution. Moreover, understanding player pacing and its relationship to the introduction of appropriate player turnover can be a valuable tool for coaches and performance staff in developing tactical strategies during match-congested schedules and making better decisions to improve team performance (20). Some reasons of appropriate player turnover could be preventing fatigue (21) or replacing injured or underperforming players (22). Thus, while it may seem counter-productive in terms of team cohesion, possessing larger squads, increasing player rotations, and having adequate substitutions could help to manage training and competition loads, potentially reducing the likelihood of injuries and, therefore, increasing player availability during match-congested schedules.

The role of the International Olympic Committee (IOC), International Federations and National Governing Bodies in responding this challenging issue (preventing fatigue and maintain performance during match-congested schedules) in sport is a key factor. For instance, the recommendation of the International Olympic Committee consensus, which indicated that soccer matches should be interspersed by at least 96 h (23, 24), has still not been taken into consideration by different sports governing bodies, which do not allow longer recovery periods between official matches than 72 h (25, 26). Moreover, some non-European soccer players belonging to European teams are required to travel to other continents (e.g., South America or Africa) to compete with their national teams, thus likely affecting their wellbeing and subsequent match performance (8). Accordingly, frequent air travel and the match-congested schedule - that are typical, for example, of the standard match schedule of the National Basketball Association (NBA) - may result in sleep disturbance diseases due to sleep length, sleep deprivation, sleep quality and sleep timing, thus resulting in highly harmful impacts on physical and mental health (27). Therefore, competitive schedules should be better organized by the sport organizations and tailored to allow sufficient time to permit sufficient recovery of the athlete, and then permitting them to maintain the high-standard level of performance or reducing the listed negative effects.

## How injuries affect performance and player availability in elite team sports?

An injury is probably the most important factor that would interfere with the readiness of an athlete to participate in competition. The cause of an injury is multifactorial and depends on intrinsic and extrinsic factors. For instance, in the NBA context, greater load and fatigue, more years of NBA experience and shorter height are associated with a higher injury risk (28). In addition, NBA schedules have been linked to in-game injury incidence but injuries occur more often in away matches (29). In soccer, it has been observed that the teams displaying good communication between the medical staff and the head coach/manager typically report a lower number of injuries and greater player availability compared with the teams with poor communication (30).

Frequently, coaches complain in the media about how the increased injury rates are affecting the competition outcome in team sport (31). For example, in the NBA there seems to be a trend between injuries and illness, and their relationship with the performance (32). In elite soccer, it has been reported a lower number of injuries is associated with a higher final league ranking, with an increased number of points per match and with an increased rate of success in the UEFA Champions League or Europa Leagues (31). Similar findings have been reported in Australian Football League teams, indicating that injury burden and player match availability are associated with final table position (33). Player availability may also affect physical performance during matches. For instance, having more soccer players injured and unavailable for match selection is associated with an increase in teams` match physical outputs (18).

Contemporary sudden stops throughout the season, such as individual COVID-19 cases and restrictions imposed by the governments to avoid the spread of COVID-19 (34), may generate new challenges on managing adequate training loads and return to training and competition (35), which can be associated to injuries and player availability. On one hand, the rate of injury in NBA players following the COVID-19 pandemic was not significantly higher during the preseason, during the first 4 weeks of the regular season or during playoffs when comparing the 2017–2018 and 2020–2021 NBA seasons (36). However, when including full seasons, there was an increased incidence of missed matches and injury ratios, from 2017 to 18 until 2020–21, even when excluding COVID-19 related cases (37). On the other hand, the Qatar 2022 FIFA World Cup is likely to be a challenge for head coaches/managers, strength and conditioning coaches, sport scientists, and medical team members of different clubs. European soccer teams without a winter break (English clubs) had a higher incidence of severe injuries following the time of the year that other European clubs which had their scheduled break (38). The absence of a winter break (i.e., a period of densely scheduled matches) could be related to insufficient physical and mental recovery with a latent cumulative fatigue, potentially contributing to poor performance and more injuries during the following period (38, 39). Additionally, it is important to assess the impact of participation in national teams at the same time as domestic and international competitions take place. In fact, the injury incidence in players participating in national team's play may not be greater than in peers who had no national obligations, but only if the coaching staff considered prior national duties when selecting, and especially substituting, players (40).

## Benefits of higher player availability in managing fatigue effects

The relevance of decision making to practice is something that is likely to resonate with sport coaches. This is evident in, for example, team selection, managing competition performance, devising strategy, managing the delivery of interventions, planning, responding to crises, providing appropriate feedback, and interacting with athletes (41). However, selecting players for the subsequent matches is perhaps one of the most important decisions that team sport coaches must make (42, 43). In fact, this decision may have a key role in the team's success (44).

The emerging collective properties of teams cannot be assigned to any single player (4). However, high-status players are disproportionately responsible for their teams' performance because they typically receive more playing time and more opportunities to impact the outcomes of matches. Additionally, high-status players are expected to elevate the performance of their teammates (45). These players generate positive flows of interaction among teammates and help other players to reach higher performance levels (46). Consequently, keeping these high-status players' injury-free and ready to participate in competition is extremely important for optimal team performance. Moreover, coaches should consider that team sport players are able to determine and modulate their output of energy dependent on the nature of the competition (47). For instance, pacing strategies differ among interchanged and whole-match rugby league players, and between winning and losing teams (48). Similarly, players during match-congested schedules can control their activity despite increasing residual fatigue (49). Thus, coaches should be aware that high-status players could distribute their energy resources while optimizing match-running performance, and while helping teammates to reach higher performance levels.

Additionally, team sport coaches need to identify fatigued players that underperform in a match as early as possible to substitute or adapt their playing style. However, the specific rules, regulations and schedules of each team sport may also influence the coaches' decisions. For instance, a handball player performing an offensive tactical variation would affect the winning or losing status (50), but this option is not allowed by other team sports rules and regulations. During a competitive basketball match, Gómez et al. (51) reported that scoring performance was significantly and positively enhanced, particularly in the first quarter, immediately following the player's substitution. However, not all team sports rules and regulations allow an unlimited number of substitutions by players during the match. Substitutions during soccer matches can minimize or offset the effects of fatigue as substitutes may cover higher distances and perform more high-intensity actions relative to entire-match players (52). While the ability to perform high-intensity activity may represent an important factor of soccer success (53), it remains unclear whether the heightened physical output observed amongst substitutes objectively reflects a positive contribution to team success (54). Thus, it might be speculated that the higher the player availability, the higher the possibility to substitute or rotate a player due to fatigue or tactical reasons and, therefore, to impact in the team's success.

It is also important to consider that elite sport team athletes are frequently required to embark on long-haul transmeridian travel for competition purposes (e.g., Olympic Games, Super Rugby Tournament, FIBA Basketball World Cup Qualifiers) exposing them to travel fatigue and jet lag. Travel fatigue can accumulate over time (55, 56), and the misalignment of the circadian system and the reported sleep restriction with the new local time may impair not only cognitive and physical performance, but, more importantly, player health and wellbeing (57, 58). Thus, one of the main questions is, what are we doing wrong when athletes report higher levels of fatigue from traveling than from training or competition? (8).

## Practical applications to increase player availability

According to the above-mentioned concerns, player availability seems to affects performance in elite team sports. Therefore, the following practical suggestions and recommendations should be considered during the systematic long-term sports preparation with the aim to increase player availability:

1) Planning constraints during training
  The levels of fatigue, the emotional state, congested schedules, or the opponent's behavior are only a few examples of constraints which demand continuous adjustments of training plans (59). Therefore, some players may not be able to train as they should in specific moments during the season. Some suggestions that can increase players' availability during training sessions are as follows:
  – Integrating the following perspectives (60): player, teammates, opponents, and time of season. All individual components of sports preparedness must be optimized, but a homogeneous fitness condition across team players based on different playing positions should be achieved. Moreover, each opponent has different individual characteristics and collective technical-tactical styles, which need to be considered and analyzed. Additionally, each moment of the season can warrant a different level of physical and psychological conditions so the management of competitive loads, as well as rest periods, depends on the number of matches and tournaments in which players/clubs participate.
  – Identify training methodologies that help player achieve the hypothetical (but close to reality) optimal load, for instance reducing the physical load from both a physiological and neuromuscular aspect possibly positively impacting the cognitive (maybe psychological) match preparation (61).
  – Identify windows of opportunities to train. Practices, although greater in frequency, should be closely monitored for total volume and should include tactical and strategical concepts, skill-based activities and minimal conditioning sessions.
  – Seizing the opportunity of the transition period to improve different aspects of performance.
  – Including a variety of challenging constraints and training variability, which improve psycho-emotional factors (e.g., motivation, joy, well-being, and adherence), the health status of performers (62) and reduce monotony in training specificity (63).
  – Consider the plausible effects of individual training strategies at private facilities, without their team's coaching staff (64).
2) Workload Monitoring
  A primary goal of workload monitoring should be to assist and inform player/coach/manager decision making on player unavailability for training (65). In this regard, workload monitoring should be carried out as follows:
  – Creating, implementing, and establishing a personalized system of fitness, workload and well-being monitoring on a daily basis (64).
  – Complementing quantitative methods (e.g., number of actions, distance covered, acceleration/decelerations) with qualitative assessments (e.g., perceived exertion, wellness).
  – Simplifying the information when reporting, limiting it to a few key and complementary metrics (65).
  – Reporting the key metrics in an easy and understandable manner using appropriate data visualization tools.
  – Establishing possible benchmarks for athletes based on the sport-specific context (i.e., sport, playing position, period of the season, age).
3) Recovery Strategies
  Teams should dedicate and concentrate their efforts to improve the player's recovery-stress balance (66). Enhancing recovery processes after training and competition is a key point to increase player availability. A further consideration is that some players may train outside of the facility adds another dimension in trying to monitor fatigue and maximize recovery. Thus, some suggestions that can help players to be able to train and compete are:
  – Prescribing the optimal load during training sessions could be the first recovery strategy (61).
  – Taking into consideration how physiological stress and physiological and biochemical markers are affected when the same players start in sequential matches and how they differ from those who remain on the bench (67, 68).
  – Using individualized protocols based on the players' characteristics such as actual fitness level, injury history and preferences (66, 69).
  – Educating players to take responsibility for their recovery.
  – Providing players with recommendations and tools (e.g., water immersion, compression therapy devices, active recovery, match ready device, among others) for their recovery management (57, 66, 70, 71).
  – Consider that some recovery strategies preferred by players may improve their happiness while other recovery strategies may be needed to improve their wellness (72). Thus, it is important to estimate the players' acceptance as recovery may be moderated by player's belief.
4) Squad Rotation
  Squad rotation is also a key issue in coping with the high demands of contemporary training and match-play. From a practical perspective, the following suggestions should be considered:
  – Having a clear criterion to clarify when to propose player rotations (probably before and not during the matches).
  – Using players from secondary teams for potentially winnable matches.
5) Travel-Related Fatigue
  There is no research-based evidence to manage travel fatigue in athletes, and low-quality evidence exists for effective interventions to recover from jet lag in athlete populations (58). Therefore, the following practical strategies should be considered with the aim of reducing travel fatigue accumulation over time (8, 56):
  – Traveling with private charter flights when possible.
  – Avoiding making player's travel who are not going to compete.
  – Minimizing international travel for injured players who are only going to be evaluated by national medical staff.
6) Return-to-Sport
  Return-to-sport decision-making is a complex process and is often characterized by uncertainties such as re-injury risk, time pressure induced by competition schedule and social stress from coaches, families and supporters (73). An inadequate return-to-sport decision has implications for the player's health and performance and for the team and training organization. Therefore, the following key points should be taken into consideration:
  – Creating, implementing, and controlling personalized preventive and corrective training programs to protect and improve locomotor health of players (64).
  – Improving the prevention process and communication among players, coaches and medical staff, because there is often little agreement between players and coaches regarding return-to-play decisions (74).
7) Communication
  The quality of communication within head coaches/managers, strength and conditioning coaches, sport scientists, and medical team members (e.g., doctors, physicians, and physiotherapists) should also be improved to increase player availability. Some practical recommendations are:
  – Ensuring well-developed communication within an interdisciplinary team of experts with clearly defined rules, roles, and responsibilities.
  – Establishing well-developed communication with the players who are the main protagonists of success and who should be provided with appropriate professional and scientific support.
  – Adopting an effective coach leadership, communicating frequently with individuals of all staff disciplines (30). This may enhance team cohesion and increase the team efficiency.

## Conclusion

Player availability, defined as keeping players injury-free and ready to participate in competition, is extremely important in the current elite team sport scenarios, because it is related to team performance. In fact, individual player availability may be more important than each player being at their individual level of peak performance. However, this topic needs to be explored more in future research. Moreover, it is imperative to highlight the prevailing need to preserve the health and wellbeing of professional players when facing a high frequency of extremely demanding matches. Thus, efforts of head coaches/managers, strength and conditioning coaches, sport scientists, and medical team members (e.g., doctors, physicians, and physiotherapists) should really focus on strategies for optimization of player availability while minimizing factors like fatigue.
